# Trends in utilization of imaging among ophthalmic-related emergency department visits in the United States

**DOI:** 10.3389/fopht.2025.1706213

**Published:** 2026-01-21

**Authors:** Brandon Chou, Serena M. Shah, Meghana Kalavar, Jayanth Sridhar, Peter MacIntosh, Andrew G. Lee

**Affiliations:** 1 Bascom Palmer Eye Institute, University of Miami Miller School of Medicine, Miami, FL, United States; 2 Illinois Eye and Ear Infirmary, University of Illinois at Chicago (UIC) Department of Ophthalmology & Visual Sciences, Chicago, IL, United States; 3 UCLA Department of Ophthalmology, Olive View-University of California Los Angeles Medical Center, Sylmar, CA, United States; 4 Department of Ophthalmology, Blanton Eye Institute, Houston Methodist Hospital, Houston, TX, United States; 5 Department of Ophthalmology, Cullen Eye Institute, Baylor College of Medicine, Houston, TX, United States; 6 Departments of Ophthalmology, Neurology, and Neurosurgery, Weill Cornell Medicine, New York, NY, United States; 7 Department of Ophthalmology, University of Texas MD Anderson Cancer Center, Houston, TX, United States; 8 Texas A&M College of Medicine, Bryan, TX, United States; 9 Department of Ophthalmology, The University of Iowa Hospitals and Clinics, Iowa City, IA, United States

**Keywords:** computed tomography, emergency department, magnetic resonance imaging, neuroimaging, ophthalmology, trends

## Abstract

**Background:**

Advanced neuroimaging use has increased in U.S. emergency departments (EDs), including for neuro-ophthalmic conditions requiring computed tomography (CT) or magnetic resonance imaging (MRI) and their use has shifted in recent years amid changing care patterns and the COVID-19 pandemic. To evaluate these changes, this study examined national trends of CT and MRI use for eye-related ED visits from 2016 to 2022.

**Materials and methods:**

A retrospective trend study was conducted using 2016–2022 National Hospital Ambulatory Medical Care Survey (NHAMCS) data. Eye-related ED visits involving CT or MRI were identified using standardized diagnostic and procedure codes. Weighted national estimates were calculated, trends were assessed with Joinpoint regression to estimate average annual percent change (AAPC), and multivariable logistic regression identified patient- and hospital-level factors associated with imaging.

**Results:**

From 2016 to 2022, eye-related ED visits totaled 42,151,975, involving 15,580,699 advanced imaging studies. Although the number of visits involving imaging remained stable, increasing by 0.8% [2,545,867 in 2016 to 2,566,826 in 2022; Average Annual Percent Change (AAPC): -0.1%], imaging rates per 1,000 visits climbed by 21.8% (AAPC: 3.1%). CT usage declined (2,445,326 in 2016 to 2,412,225 in 2022; AAPC: -1.3%), while MRI usage rose (332,588 in 2016 to 345,153 in 2022; AAPC: 1.0%). Younger age, race (particularly black patients), and Medicaid coverage were associated with reduced likelihood of imaging, while residence in the Midwest or South increased odds. Hospital admission remained the strongest predictor, tripling the likelihood of imaging. The COVID-19 pandemic drove a notable rise in imaging rates (from 34.9% before the pandemic to 40.1%; p=0.003).

**Conclusion:**

Advanced imaging use during eye-related ED visits increased from 2016 to 2022 with a shift toward greater MRI utilization and stable CT usage. Demographic factors and the COVID-19 pandemic potentially contributed to these trends and observed racial disparities highlight potential systemic barriers to imaging access. Despite limitations related to hospital representation and data scope, these findings emphasize the need for further research to explore drivers of imaging use and address healthcare inequities.

## 1 Introduction

The use of advanced diagnostic imaging, including computed tomography (CT) and magnetic resonance (MR) scans in the United States (US) has grown over the last two decades (1–3). This trend has been observed across multiple clinical settings with emergency departments (EDs) accounting for a substantial portion of imaging growth (1, 2). In ophthalmology, a similar trend has been observed in the ED for neuro-ophthalmic conditions which frequently necessitate advanced diagnostic imaging (CT and or MRI) to evaluate intracranial, orbital, or optic nerve pathology (4, 5). Although appropriate imaging can guide diagnosis and treatment, overuse contributes substantially to rising healthcare costs, with unnecessary imaging estimated to add $200–250 billion annually to the U.S. healthcare system (6). Within ophthalmology, studies have also highlighted both the increased frequency and cost of excess imaging (5, 7, 8).

Concerns about the appropriateness and poor practices of neuroimaging for eye-related complaints are also well-documented (9). At one academic neuro-ophthalmology practice, referrals of new patients who had undergone neuroimaging (n=84) resulted in 38.1% (n=32) of suboptimal neuroimaging studies; consequently, additional neuroimaging was required for 24 of 84 subjects (28.6%) (9). Additionally, overuse of imaging can also expose patients to ionizing radiation (with CT) and incidental findings that may complicate care (10, 11).

EDs represent a key setting for advanced imaging in patients with acute eye-related complaints (5, 12, 13). The COVID-19 pandemic further altered ED utilization of imaging, with many centers reporting an overall decline in ED volumes but a relative increase in advanced imaging for patients presenting with acute visual or neurologic complaints, likely due to concerns of more severe manifestations of disease resulting in ED visits (14–16). While earlier studies have described national trends in ophthalmic imaging within the ED, few have examined these patterns in recent years or accounted for the impact of the COVID-19 pandemic at a national level, which may have further influenced imaging practices and healthcare utilization (5, 14, 17).

Against this backdrop of rising utilization, financial implications, and ongoing concern about appropriateness, understanding contemporary imaging patterns is important for potentially guiding clinical practice and policy discussions. To characterize how imaging use has evolved in recent years, this study examines nationwide trends and determinants of CT and MRI use during eye-related ED visits in the United States from 2016 to 2022 using data from the National Hospital Ambulatory Medical Care Survey (NHAMCS).

## 2 Methods

The NHAMCS, conducted by the National Center for Health Statistics (NCHS), is a nationally representative database that collects information about visits to emergency departments at nonfederal, general, and short-stay hospitals in all 50 states and the District of Columbia. Federal, military, and veterans administration hospitals are excluded (18). Data about the emergency department visit is obtained and organized based on patient characteristics, such as age, sex, race, and ethnicity, visit reason, provider’s diagnosis, services provided, treatment provided, and more (19). Because NHAMCS data are publicly available and de-identified, this study was exempt from institutional review board oversight.

Our study is a retrospective analysis of CT and MR scan use in patients in the US with visits to the ED involving eye problems between 2016 and 2022. Cases involving eye problems were identified by referencing the American Academy of Ophthalmology (AAO) quick reference guides for ophthalmic International Classification of Diseases, Tenth Revision, Clinical Modification (ICD-10-CM) codes as of October 1, 2022, and applying a filter to the NHAMCS database to select for those codes (20). A full list of codes are provided in the **Supplementary Materials**. Because the NHAMCS includes up to 3 diagnoses for each patient visit, only ED visits where the principal or first diagnosis was an eye-related problem were selected for inclusion.

Results were weighted to create estimates that were nationally representative for each individual year and the entire 7-year period according to the sampling structure of the NHAMCS which in brief consists of survey visit weights (PATWT) (18). NHAMCS includes standardized National Center for Health Statistics (NCHS) imputations for age, sex, race, and ethnicity, along with masking and top-coding. Missingness in variables used for this analysis was minimal, and all NCHS-provided imputations were retained without additional imputation. Further details on imputation procedures are available in the NHAMCS documentation.

Annual estimates of total eye-related ED visits were calculated, and then the percentage of those associated with CT or MRI imaging was calculated. Total eye-related ED visits associated with CT or MRI imaging were then stratified by patient and institute-based characteristics. The average annual percent change (AAPC) in imaging utilization, derived from estimating an underlying Joinpoint model that best fits the data using associated weighted averages of slope coefficients, was calculated. This technique has been used in imaging trend studies documented in the literature (21, 22). More specifically, annual percentage change (APC) is a measure that is calculated using a logarithmic regression model that accounts for the fact that the rate of change in imaging between years can differ. This technique allows multiple APCs to be calculated and the creation of a “Joinpoint” to delineate between APCs; AAPC is a weighted average of APCs according to the time interval of the trend in APCs.

Medicare reimbursement rates for CT and MRI were obtained using the Centers for Medicare and Medicaid Services Physician Fee Schedule for each study year from 2016 to 2022 (23). Facility-based payments and fully implemented facility total RVUs were collected for the relevant CPT codes for CT, 70450, 70460, and 70470 and MRI, 70551, 70552, and 70553. To adjust for inflation, the consumer price index (CPI) inflation calculator from U.S. Department of Labor’s Bureau of Labor Statistics was used (24). To reflect overall reimbursement rather than any single geographic locality, national default values were used. MRI codes were combined into a single MRI category and CT codes into a single CT category as an average because NHAMCS does not detail what exact imaging was performed. For each year and modality group, the mean facility payment was calculated as the average cost per scan across the study period.

Descriptive analyses with chi-square comparisons of all study measures were performed to assess the proportion of visits receiving imaging, and a multivariate logistic regression was created to identify factors that were independently associated with higher likelihood of imaging use. Model diagnostics included variance inflation factors (VIFs) to assess multicollinearity and model performance was assessed with McFadden and Nagelkerke pseudo-R² values. All p values < 0.05 were considered significant and statistical analyses were conducted using a combination of R Version 4.2.3, SPSS Version 28, and Joinpoint Version 5.0.2.

## 3 Results

### 3.1 Overall imaging trends

**Figure 1:**
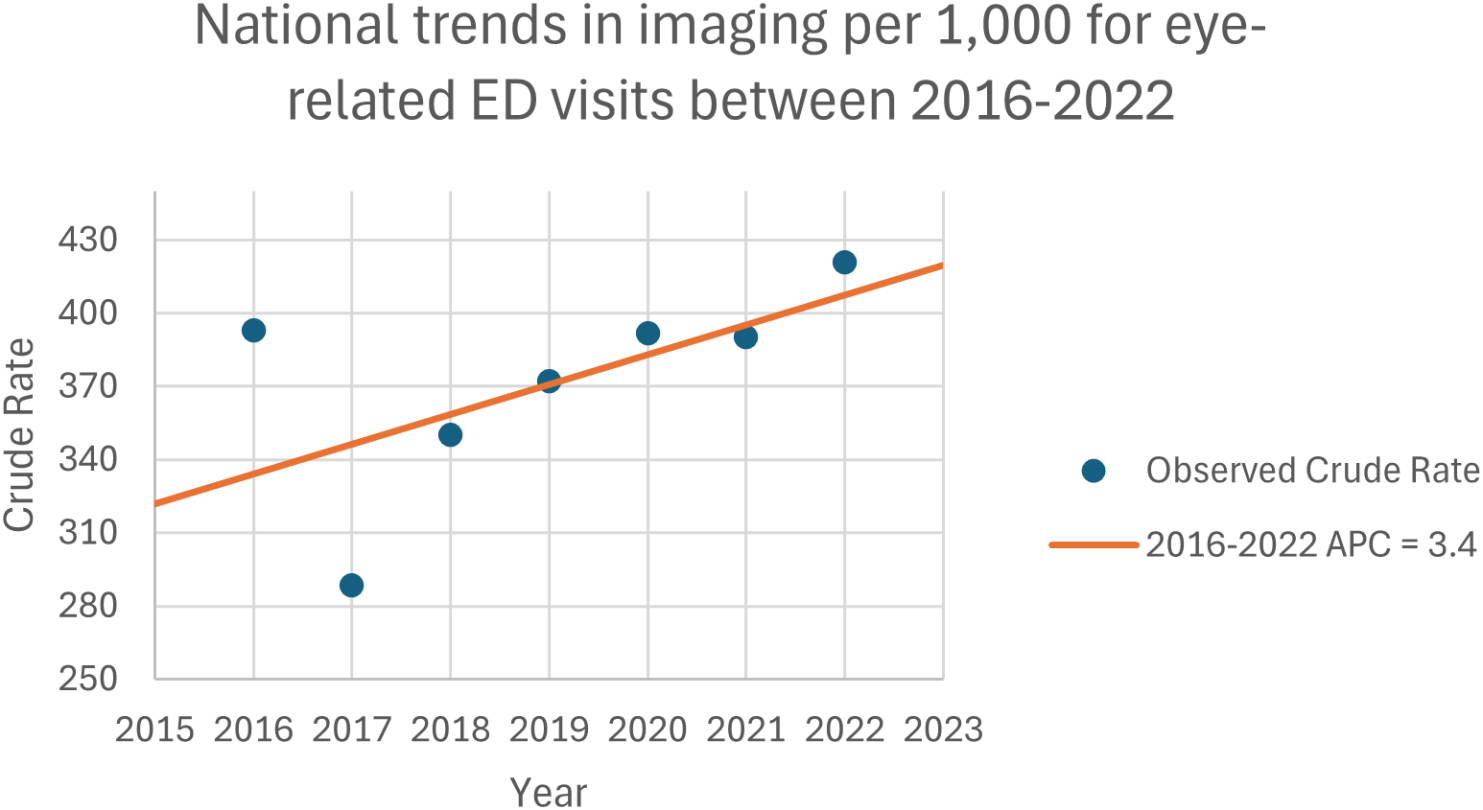
National trends in imaging rates per 1,000 for eye-related emergency department visits between 2016 and 2022.

Between 2016 and 2022, a total of 15,580,699 CT and MR scans were performed during 42,151,975 eye-related ED visits. While the total number of eye-related ED visits involving CT and MR scans remained mostly consistent, increasing by 0.8% over the study period (from 2,545,867 in 2016 to 2,566,826 in 2022, AAPC: -0.1%), the rate of imaging per 1,000 visits rose by 21.8% (from 335.3 to 408.5), with an AAPC of 3.1% (**Table 1**, **Supplementary Table S1**, **Figure 1**).

**Table 1:** Demographics of patients who underwent CT or MRI vs those who did not undergo imaging for eye ED visits between 2016-2022.

Patient characteristics	Characteristics of patients who underwent imaging and had an eye visit, n (%)	Characteristics of patients who did not undergo imaging and had an eye visit, n (%)	p-value
Age categories			<0.001
<15	584,335 (3.8)	3,662,196 (13.8)	
15 ≤ 24	1,057,644 (6.8)	3,836,217 (14.4)	
25 ≤ 44	3,238,719 (20.8)	8,490,564 (32.0)	
45 ≤ 64	5,109,888 (32.8)	6,585,457 (24.8)	
65 ≤ 74	2,478,286 (15.9)	2,073,568 (7.8)	
≥75	3,111,828 (20.0)	1,923,275 (7.2)	
Sex			<0.001
Female	9,419,881 (60.5)	15,598,620 (58.7)	
Male	6,160,818 (39.5)	10,972,656 (41.3)	
Race			<0.001
White	11,570,659 (74.3)	18,015,077 (67.8)	
Black	3,175,340 (20.4)	7,504,961 (28.2)	
Other	834,699 (5.4)	1,051,237 (4.0)	
Region			<0.001
Northeast	2,438,328 (15.6)	4,699,826 (17.7)	
Midwest	3,518,229 (22.6)	5,694,955 (21.4)	
South	6,206,106 (39.8)	9,707,991 (36.5)	
West	3,418,036 (21.9)	6,468,504 (24.3)	
Insurance status			<0.001
Private	3,878,594 (24.9)	6,804,227 (25.6)	
Medicare	5,461,215 (35.1)	4,331,243 (16.3)	
Medicaid	3,045,166 (19.5)	9,127,134 (34.3)	
Other	3,195,725 (20.5)	6,308,672 (23.7)	
Day of visit			<0.001
Weekend	3,756,179 (24.1)	6,709,392 (25.3)	
Weekday	11,824,520 (75.9)	19,861,883 (74.7)	
Admission status			<0.001
Not admitted	12,874,631 (82.6)	25,423,304 (95.7)	
Admitted	2,706,068 (17.4)	1,147,971 (4.3)	
Population			<0.001
Urban	13,725,395 (88.1)	23,335,618 (87.8)	
Rural	1,855,304 (11.9)	3,235,658 (12.2)	
ED residency			<0.001
Yes	4,570,678 (31.1)	8,943,491 (36.2)	
No	10,114,735 (68.9)	15,771,474 (63.8)	
Healthcare provider seen			<0.001
ED attending physician	14,480,263 (92.9)	22,336,773 (84.1)	
ED resident/intern	2,118,615 (13.6)	3,512,652 (13.2)	
Consulting physician	3,132,140 (20.1)	2,506,248 (9.4)	
Physician assistant	2,112,374 (13.6)	4,191,885 (15.8)	
Nurse practitioner	1,558,288 (10.0)	2,820,236 (10.6)	

### 3.2 Imaging modality

CT scan remained a predominant modality. Between 2016 and 2022, the number of CT scans performed during eye-related ED visits decreased overall (AAPC -1.3%), with two distinct trends: decreasing from 2016-2018 (APC -12.7%) and increasing from 2018-2022 (APC 4.9%). CT rates per 1,000 visits (from 318.6 to 380.0) increased by 19.3% (AAPC 3.0%). Over the 7-year study period, the most frequent diagnoses associated with CT imaging included headache (27.1%), dizziness and giddiness (22.1%), and unspecified headache (11.8%). Other diagnoses are detailed in **Table 2**, including unspecified cerebral infarction (11.4%), unspecified transient cerebral ischemic attack (7.6%) and Bell’s palsy (2.1%).

**Table 2:** Overall annual imaging use based on ICD-10-CM diagnoses.

Diagnosis	2016	2017	2018	2019	2020	2021	2022
Headache (R51-) Total visits	2,135,994	1,918,406	1,872,832	2,121,842	1,882,333	1,760,324	2,041,300
Headache (R51-) Visits with imaging	978,011	737,978	704,138	999,865	779,238	758,602	1,036,595
Dizziness and giddiness (R42-) Total visits	1,346,983	932,446	1,018,547	1,317,800	1,096,593	1,209,403	1,311,478
Dizziness and giddiness (R42-) Visits with imaging	575,661	349,238	489,252	553,978	438,839	541,076	473,641
Cerebral infarction, unspecified (I639) Total visits	278,665	288,553	268,954	181,402	417,092	304,739	443,780
Cerebral infarction, unspecified (I639) Visits with imaging	234,388	252,369	218,608	123,939	344,168	297,070	350,168
Transient cerebral ischemic attack (G459) Total visits	260,255	127,176	159,225	247,653	119,999	164,314	233,211
Transient cerebral ischemic attack (G459) Visits with imaging	260,255	88,761	134,617	210,427	115,826	123,600	221,407
Bell’s palsy (G510) Total visits	148,597	103,198	93,791	145,968	52,162	42,868	79,304
Bell’s palsy (G510) Visits with imaging	63,624	50,995	36,569	64,786	30,550	19,339	64,937

In contrast, the number of MR scans showed an overall increase over the same period (AAPC 1.0%), again with two distinct trends: increasing from 2016-2020 (APC 5.0%) and decreasing from 2020-2022 (APC -6.7%). MR scan rates per 1,000 visits grew moderately over the study period (from 48.1 to 61.0) (AAPC 4.0%) with corresponding increasing trend from 2016-2020 (APC 11.5%) and decreasing trend from 2020-2022 (APC -9.5%). The primary diagnoses for which MR scans were performed during this 7-year period were unspecified cerebral infarction (24.1%), dizziness and giddiness (21.0%), headache (14.5%), and unspecified transient cerebral ischemic attack (10.6%).

### 3.3 Patient demographics and visit characteristics

Differences in characteristics between eye-related ED visits that involved imaging versus those that did not are summarized in **Table 1**. Across the 7-year study period, the patient population demonstrated a broad age distribution, with adults aged 25–44 years (20.9%) and 45–64 years (32.8%) comprising the largest proportions of visits overall. Females accounted for the majority of patients (60.4%), and white individuals represented the largest racial group (74.3%).

Imaging use varied by age group (**Supplementary Table S2**). Among older adults (≥75 years), the proportion undergoing imaging decreased from 68.0% to 66.8%, however the overall trend has been increasing (AAPC 2.1%). In contrast, imaging use among children younger than 15 years remained low, peaking at 18.6% before declining to 13.2%, although the overall trend too has been increasing (AAPC 1.9%).

Overall imaging rates by gender remained relatively stable during the study period, with a dip in 2017 followed by an increase in subsequent years. Females consistently had higher imaging rates than males, peaking at 436.3 per 1,000 visits in 2022 (AAPC 3.2% for females versus 3.6% for males); however, male imaging rates eventually surpassed female rates by 2020 (40.0% for males versus 39.0% for females) but did not hold into 2022 (40.0% for males versus 43.6% for females). This pattern held for MR imaging but not for CT scans, where males had higher utilization of MR imaging in 2022 (6.5% for males versus 5.0% for females) (**Table 3**).

**Table 3:** Overall imaging use based on gender.

Imaging type	2016	2017	2018	2019	2020	2021	2022
Overall imaging (%)							
Female	39.4	29.4	35.5	41.0	39.0	37.5	43.6
Male	39.1	27.9	34.3	32.0	39.5	41.3	39.9
CT imaging (%)							
Female	38.9	28.0	33.2	37.6	36.5	36.3	40.6
Male	36.2	26.8	32.7	28.8	35.7	38.2	38.0
MRI imaging (%)							
Female	3.8	4.8	6.2	6.0	5.7	8.3	5.0
Male	7.0	4.9	6.6	6.3	9.8	7.1	6.5

Imaging rates among white patients followed a similar pattern, with a temporary decline in 2017 and steady increases from 2020 onwards. CT and MR scan use per 1,000 visits remained higher among white patients (424.9 in 2022, AAPC 2.7%) than black patients (320.9 in 2022, AAPC 2.6%), with utilization increasing more markedly among white patients (**Table 4**). In contrast, imaging rates among black patients remained lower overall and relatively unchanged through 2021 before increasing to 37.8% in 2022 rising after remaining near 27.0% from 2016-2021. This pattern of higher and increasing imaging rates for white patients compared with black patients was consistent across CT and MRI modalities (**Table 4**).

**Table 4:** Overall imaging use involving race.

Imaging type	2016	2017	2018	2019	2020	2021	2022
Overall imaging (%)							
White	41.0	32.4	37.1	38.4	41.6	42.5	42.4
Black	34.9	21.5	27.6	33.1	28.8	27.0	37.8
Other	29.5	6.7	49.8	45.8	51.3	46.7	53.5
CT imaging (%)							
White	39.7	30.7	35.5	34.2	37.7	40.2	39.3
Black	33.1	21.2	24.8	32.5	27.5	25.7	37.5
Other	24.1	6.7	47.0	38.5	50.7	46.7	48.9
MRI imaging (%)							
White	5.1	5.2	7.1	7.3	6.7	8.5	5.9
Black	4.1	4.3	4.0	2.5	2.6	6.2	3.8
Other	13.9	No data	10.5	11.5	28.6	5.7	9.6

### 3.4 Insurance and hospital setting

Medicare (35.1%) was the most common payer among eye-related ED visits involving imaging, followed by private insurance (24.9%), Other (20.5%), and Medicare (19.5%). Imaging rates per 1,000 visits increased among patients with Medicaid (268.1 in 2022, AAPC 2.4%) and private insurance (369.6 in 2022, AAPC 1.0%) but declined among Medicare beneficiaries before increasing from 2020 onwards (AAPC 4.3%) (**Supplementary Table S3**).

Urban hospitals accounted for the majority of imaging procedures (88.1%). Both urban and rural hospitals saw moderate increases in imaging use per 1,000 eye-related ED visits, with urban hospitals showing a slightly higher AAPC except during 2020 in which rural hospitals had an observed rate of 473.8 per 1,000 visits compared to those of urban hospitals with 385.6 per 1,000 visits (384.2 in 2022, 2.0% for rural hospitals versus 411.2 in 2022, 3.5% for urban hospitals). Hospitals without an emergency medicine residency program demonstrated greater increases in imaging use compared with those with one (AAPC 2.5% for those with an emergency medicine program vs 4.1% for those without) (**Supplementary Table S4**).

### 3.5 Diagnostic reasons for eye-related ED visits

**Figure 2:**
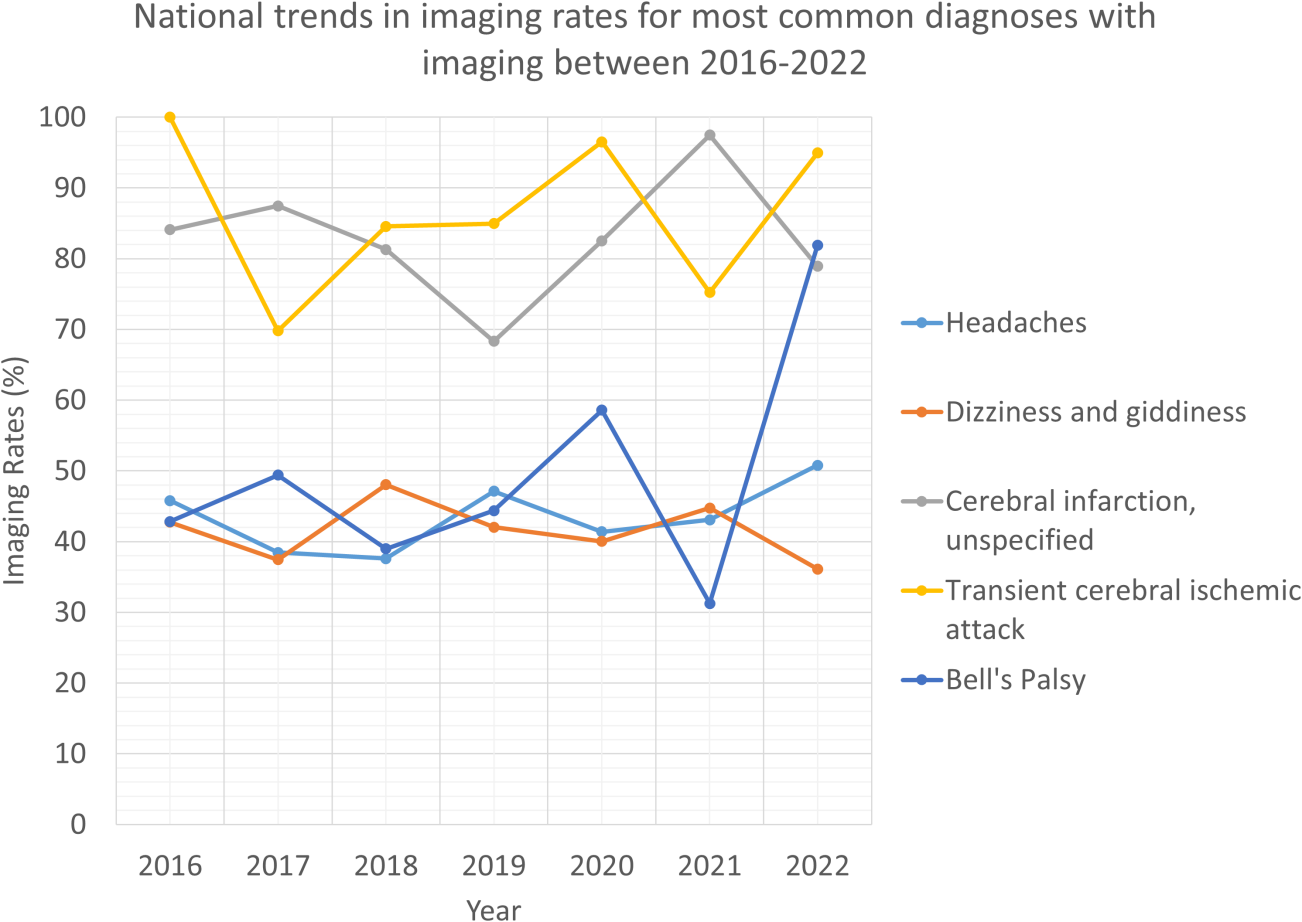
National trends in imaging rates for most common diagnoses associated with imaging between 2016 and 2022.

ICD-10 codes for headache, dizziness and giddiness, and cerebral infarction unspecified were the most frequent diagnoses necessitating imaging during eye-related ED visits (**Table 2**, **Figure 2**). Of note, more than 70% of visits that involved imaging had associated central nervous system diagnoses, underscoring the overlap between eye-related complaints and neurologic conditions. Across both CT and MRI modalities, the most common diagnostic reasons for imaging were headache, dizziness and giddiness, and cerebral infarction, which together accounted for more than 80% of all imaging performed during eye-related ED visits (**Table 2**). Imaging for Bell’s palsy, though less common overall, showed the largest relative increase (AAPC 5.3%), whereas imaging for dizziness and giddiness declined (AAPC -1.2%).

### 3.6 Multivariable analysis

A multivariable model was also constructed which demonstrated modest explanatory power, with a McFadden pseudo-R² of 0.10 and a Nagelkerke pseudo-R² of 0.17. We also assessed multicollinearity among all independent variables. Variance inflation factors for predictors ranged from approximately 1.0 to 1.6, indicating no concerning multicollinearity and supporting the stability of the multivariable estimates.

On multivariable analysis via a logistic regression model, older age remained significantly associated with higher odds of receiving advanced imaging during an eye-related ED visit. Sex, rural versus urban location, weekend presentation, and most insurance types were not independently associated with imaging. Compared with white patients, Black patients had significantly lower odds of undergoing imaging, while patients categorized as other race did not differ significantly. Imaging rates varied by geographic region: visits in the Midwest and South had higher odds of imaging compared with those in the Northeast, whereas visits in the West were similar. Patients covered by Medicaid had lower odds of imaging relative to those with private insurance. Hospital admission was the strongest predictor of imaging use; admitted patients were approximately three times as likely to undergo CT or MRI as non-admitted patients. Full odds ratios and confidence intervals are provided in **Table 5**.

**Table 5:** Multivariate logistic regression model predicting the likelihood of having CT or MRI during an eye ED visit, 2016-2022.

Variable	Odds ratio	95% confidence interval	p-value
Age (continuous)	1.028	1.022-1.033	<0.001
Sex (reference: female)			
Male	0.932	0.794-1.095	0.393
Race (reference: white)			
Black	0.769	0.625-0.946	0.013
Other	1.228	0.904-1.669	0.190
Location (reference: urban)			
Rural	0.855	0.687-1.064	0.161
Geographic region (reference: Northeast)			
Midwest	1.396	1.032-1.889	0.031
South	1.416	1.065-1.881	0.017
West	1.009	0.747-1.361	0.956
Insurance type (reference: private)			
Medicare	0.876	0.694-1.106	0.265
Medicaid	0.761	0.612-0.948	0.015
Other	0.863	0.661-1.128	0.282
Day of visit (reference: weekday)			
Weekend	0.955	0.798-1.143	0.616
Admission status (reference: not admitted)			
Admitted	3.036	2.155-4.278	<0.001

### 3.7 Impact of the COVID-19 pandemic

Imaging rates increased after the first year of the COVID-19 pandemic (2020). Before the pandemic (2016-2019), 34.9% of eye-related ED visits involved imaging, compared with 40.1% from 2020 onwards (p=0.003). CT imaging increased from 32.9% pre-pandemic to 37.7% (p=0.010) from 2020 onwards, while MRI imaging rose from 5.6% to 6.9% (p=0.200).

### 3.8 Annual imaging costs

Inflation-adjusted facility costs for CT and MRI during eye-related ED visits demonstrated gradual declines in mean cost per scan between 2016 and 2022 (**Table 6**). The mean CT facility payment decreased from $66.04 in 2016 to $52.83 in 2022, while the mean MRI facility payment declined from $112.48 to $90.21 over the same period. Despite these reductions in per-scan costs, resulting in decreased national CT-related facility costs and MRI-related facility costs, total national spending for advanced imaging during eye-related ED visits remains substantial due to high imaging volume.

**Table 6:** Annual CT and MRI use and estimated facility costs for eye-related ED visits, 2016-2022.

Year	CT visits	MRI visits	CT mean cost per scan*	MRI mean cost per scan*	CT total cost*	MRI total cost*
2016	2,445,326	332,588	66.04	112.48	161,482,163	37,410,369
2017	2,030,787	357,870	64.53	110.05	131,040,448	39,383,348
2018	1,804,115	348,723	62.92	107.26	113,512,055	37,403,892
2019	2,110,873	381,106	62.43	106.29	131,779,265	40,508,407
2020	2,002,971	405,230	60.45	103.46	121,075,147	41,924,760
2021	1,843,877	389,095	56.87	97.20	104,868,794	37,818,134
2022	2,412,225	345,153	52.83	90.21	127,437,866	31,135,085

*Costs are inflation-adjusted facility payments in 2022 dollars based on national reimbursement. CT costs are averaged from the following CPT codes: 70450, 70460, and 70470. MRI costs are averaged from the following CPT codes: 70551, 70552, and 70553.

## 4 Discussion

Overall, the NHAMCS data revealed increases in combined CT and MR scan rates rather than changes in total volume among eye-related ED visits from 2016 to 2022. These trends may reflect evolving diagnostic practices from the distinct utilities of CT and MRI for various pathologies, alongside regional, racial, and socioeconomic disparities. The rise in imaging rates during the COVID-19 pandemic, despite stable visit numbers, may also reflect shifts in emergency care delivery during this period. Despite how mean facility payments for CT and MRI declined over time, the large volume of imaging during eye-related ED visits means that aggregate spending remains substantial, underscoring the financial implications of small changes in imaging rates. Although NHAMCS does not fully capture clinical indications or imaging appropriateness, these results offer a nationally representative assessment of imaging utilization and where notable disparities, or temporal shifts have emerged.

When stratifying the overall imaging trends by modality, we observed an initial decrease followed by an increase in CT utilization among eye-related ED visits from 2016 to 2022 with an inflection point in 2018 resulting in a small net decline across the study period. Given the limitations of NHAMCS, we cannot determine the underlying clinical motivations for these shifts directly, however this observation may partly reflect a growing recognition among clinicians that CT is a less valuable diagnostic tool for many ophthalmic conditions compared to MRI. Clinicians have continued to rely on CT in acute settings involving ophthalmic conditions with neurological origins, as CT enables rapid imaging of static central nervous system (CNS) structures contributing to the visual system, such as the optic nerve and occipital lobe (4). CT is also recognized as a valuable tool for evaluating ocular trauma, particularly in cases involving potential orbital fractures (25). These insights support our findings that the most common diagnoses most frequently associated with CT imaging in our dataset included headache, dizziness and giddiness, cerebral infarction unspecified, transient cerebral ischemic attack, Bell’s palsy, and cerebral infarction which are diagnoses whose differentials include conditions that require acute care such as brain hemorrhage and stroke. However, in cases where ophthalmic issues do not involve neurologic or traumatic etiologies, clinicians may in some settings perceive CT as less diagnostically useful, which could explain the initial decreased rates of CT utilization we observed in our study (5). Although taken together, the early decrease in CT rates followed by later increase may simply reflect fluctuations in the ED or changes in institutional imaging workflows rather than a broad shift away from CT.

Conversely, we observed an increase in MRI utilization followed by a decrease with an inflection point in 2020 with a net increase in utilization rates. This observation may be consistent with the general impression among ophthalmologists that MRI provides superior visualization of soft tissue orbital and intracranial tissue, such as the optic nerve, although NHAMCS cannot confirm that these clinical considerations drove the observed trends (26, 27). Over our study period, providers most commonly used MRI to evaluate cases involving cerebral infarction unspecified, headache, and dizziness and giddiness which are ocular symptoms frequently linked to neurologic origins. Experience from one ophthalmology-specific ED further highlights this trend in which MRI use nearly tripled CT and was most often ordered for neuro-ophthalmic indications such as optic disc edema, diplopia, and cranial nerve palsy (28). Notably, the increasing MRI utilization we observed at a national level compared to the findings reported by Zafar may suggest a gradual shift in general ED practice patterns toward those seen in ophthalmology-specific EDs, potentially reflecting broader recognition of MRI’s higher diagnostic yield in neuro-ophthalmic disease or wider availability of this modality (5, 28). In our study, admitted patients also had markedly higher odds of undergoing imaging, yet only 17.4% of eye-related ED visits with imaging resulted in hospital admission, indicating that most scans are performed in patients who are ultimately discharged. This pattern suggests that advanced imaging is frequently being used in patients who do not require admission, although NHAMCS cannot assess diagnostic yield or downstream clinical benefit.

Several broader epidemiologic trends could plausibly intersect with the MRI utilization patterns we observed, although NHAMCS does not include diagnosis-level detail sufficient to link these conditions directly to imaging use. For example, Hassan et al. documented a steady rise in orbital tumor incidence in the U.S. through 2009, suggesting this trend may have continued through 2020 with MRI serving as a key tool for evaluating orbital tumors (29). Patients over age 60 frequently also develop lymphoproliferative lesions as orbital tumors, which may contribute to the elevated imaging rates we found among those aged 75 and older (30–32). Changing tobacco use patterns may also play a role; although traditional cigarette smoking has declined, e-cigarette use has grown, and both are associated with recurrent orbital inflammation and conditions such as thyroid eye disease that may necessitate neuroimaging (33–35). Additionally, MRI is commonly used to evaluate optic neuritis and related conditions like multiple sclerosis, yet nearly 60% of patients referred for “optic neuritis” are ultimately diagnosed with alternative conditions, suggesting that diagnostic uncertainty may drive more frequent imaging (36). Rising rates of cavernous venous sinus thrombosis (CVST) in the U.S., as reported by Otite et al., may also contribute, especially since Wang et al. showed that a substantial portion of CVST patients present with ocular symptoms, which typically prompts MRI evaluation (37, 38). Together, these factors may help contextualize the rising imaging rates especially with respect to increased MRI usage that we observed between 2016 and 2020, however these examples are provided as potential clinical contexts rather than as direct explanations as the NHAMCS cannot resolve diagnosis-level drivers of imaging.

When examining gender disparities, we found that instances of imaging among patients presenting to the ED for eye-related problems were higher in females than in males. This trend may reflect the well-documented pattern that females utilize healthcare services more frequently than males (39, 40). For instance, the 2013 Kaiser Men’s Health Survey and Women’s Health Survey, which included a nationally representative sample of U.S. adults aged 18 to 64, showed that 81% of women sought routine care from a clinician compared to 68% of men (39). Additionally, Weiss et al. reported that in 2018, females had a higher overall rate of ED visits than males (40). However, when analyzing rates within gender in our study, rates of imaging among males overtook that of females by 2020 in which males demonstrated growth in CT imaging rates and exceeded in MR scan rates (9.8%) compared to that of females (5.7%). Interestingly, in our study, imaging rates among male patients peaked in 2020, coinciding with the onset of the COVID-19 pandemic although this trend did not hold for CT into 2022 but did for MR imaging. This reversal should therefore be interpreted cautiously and may reflect pandemic-specific change. Although this pattern does align with broader ED trends during that time, as Heppner et al. found that patients presenting to the ED during the pandemic were more likely to be male, arrive by ambulance, and present with toxicology, psychiatric, or infectious disease pathologies (41). Similar trends were seen at an eye-specific ED that observed an overall decrease in median number of daily visits to the ED with a concurrent 9% increase in primary diagnoses considered urgent (p=0.0002) and 29% increase in the proportion of visits requiring urgent surgery (p=0.004) between 2018-2020; the study did not stratify by gender of the visits considered urgent or of those requiring surgery (14). Moreover, since men with COVID-19 were more likely to experience severe outcomes or death than women, this could have led to an increased need for head imaging in this population, although NHAMCS does not contain granularity to allow for this attribution (41).

Racial disparities were pronounced in our results, as white patients consistently received more imaging compared to black patients. This disparity widened notably during the pandemic, with imaging rates exceeding 40% for white patients but remaining below 30% for black patients. These findings were consistent to those reported by Arnett et al. who found that although black individuals were more likely than white individuals to use the ED and hospital outpatient departments as a usual source of care, providers may still image white patients more frequently during these visits (42). This pattern could explain the disparity in imaging between white and black patients in our study. Other studies utilizing the NHAMCS dataset for all imaging modalities found a similar trend, that while controlling for other patient and hospital characteristics, imaging rates of white patients exceeded that of non-white patients significantly in all imaging modalities except for ultrasound (43). In a review on imaging disparities in radiology by Waite et al., potential reasons posed included patient-related factors of health literacy, medical mistrust, cultural differences, and communication barriers such as lack of awareness of options of medical screening. System-related factors include disproportional care received at lower-quality hospitals which may not readily have access to newer imaging modalities, and race-based diagnostic algorithms which may direct more attention and resources to white patients (44). Although NHAMCS does not allow for ascertaining social determinants well, taken together, these findings potentially suggest that both patient-level and systemic factors may contribute to the persistent racial disparities in imaging utilization observed in ophthalmic-related ED visits and warrant further investigation.

Geographic and hospital-level patterns also warrant consideration. In our multivariable model, imaging odds were higher in the Midwest and South compared with the Northeast, while the West was similar. Rural hospitals had slightly lower overall imaging rates but experienced sharper increases during the early pandemic. Prior NHAMCS work by Dubey et al. demonstrated that geographic variation in imaging use has been longstanding, with significantly higher odds of head CT in the Midwest (OR 1.27, 95% CI 1.12-1.44) and the South (OR 1.19, 95% CI 1.07-1.33) compared with the Northeast, while the West showed no meaningful difference (OR 0.94, 95% CI 0.83-1.06) (21). Rural hospitals in their analysis likewise demonstrated lower overall odds of imaging (OR 0.85, 95% CI 0.77-0.94) (21). Limited technical capacity is unlikely to fully explain these patterns. A national survey found that 96% of rural EDs had on-site CT access, suggesting that disparities may reflect practice norms, physician preference, or workflow differences rather than equipment availability alone (45). Additionally, 70% of rural ED physicians completed residency twenty or more years ago, indicating that training-era differences may shape diagnostic habits and contribute to regional variation (46). Although NHAMCS cannot resolve the underlying drivers of these trends, these data highlight the need for future work examining how local workforce characteristics, clinical culture, and resource use patterns may contribute to geographic disparities in imaging.

Following the onset of the COVID-19 pandemic in 2020, our results show that imaging utilization during eye-related ED visits increased significantly, potentially due to worsening neuro-ophthalmic conditions from reduced specialist access and potential neuro-ophthalmic manifestations of COVID-19, although NHAMCS cannot directly assess this attribution. This trend aligns with the report by Waisberg et al. that during the COVID-19 pandemic, neuro-ophthalmic diseases, such as idiopathic intracranial hypertension, compressive optic neuropathy, optic neuritis, and giant cell arteritis, all worsened possibly due to the lack of regular neuro-ophthalmology follow up. Perhaps these neuro-ophthalmology patients ended up in ED settings with worsened disease, necessitating imaging (16). Additionally, direct neuro-ophthalmic manifestations of COVID-19, including cranial nerve palsies and optic neuritis may have further contributed to increased imaging demands during the pandemic (47). Overall, between pre-existing neuro-ophthalmic conditions and the direct effects of COVID-19, both of these factors likely contributed to the rise in imaging utilization during eye-related ED visits observed in our study, highlighting the pandemic’s broad impact on emergency ophthalmic care, but these proposed mechanisms should be regarded as conjectural and are offered as clinical context rather than empiric findings from NHAMCS.

Several factors may have influenced our study. Most notably, ED settings from federal, military, and Veterans Health Administration hospitals are excluded from NHAMCS and therefore, conclusions about data points from these settings and how they might have impacted the results of our project cannot be stated. Other limitations include misdiagnosis of ICD codes and inability to identify specifically COVID-associated diagnoses. This limitation applies to other factors posed by NHAMCS such as how hospital and survey staff classify patient race and ethnicity, how the study has no direct measure of socioeconomic status, and the nature of the NHAMCS as a national estimate in which one cannot ascertain individual patient level data. Furthermore, the multivariable model explained a modest proportion of variability in imaging use (McFadden pseudo-R² 0.10; Nagelkerke pseudo-R² 0.17), indicating that many clinical and institutional factors influencing imaging decisions are not captured in NHAMCS. Variance inflation factors were low, suggesting that collinearity among included predictors was not a major concern; nonetheless, the modest explanatory power highlights the importance of viewing the regression results as one piece of a broader descriptive picture rather than a comprehensive predictive model. Finally, appropriateness of imaging cannot be determined and remains an important dimension of imaging use as well. The temporal gap between data collection, which ended in 2022 with the discontinuation of the NHAMCS, and current clinical practice represents a limitation especially given evolving ED imaging stewardship initiatives and ongoing post-pandemic shifts.

In conclusion, our study found increases in rates of imaging utilization during eye-related ED visits from 2016 to 2022, associated with factors such as race, other socioeconomic factors, and the COVID-19 pandemic. The rise in MRI usage and the stagnation in CT usage may reflect evolving diagnostic preferences, particularly for neuro-ophthalmic conditions. Racial disparities in imaging utilization suggest systemic factors influencing healthcare access. Although NHAMCS lacks sufficient detail to apply decision frameworks of imaging appropriateness at the visit level, our findings identify patient and hospital groups where imaging is concentrated or comparatively underused, which may help prioritize future work that can directly evaluate guideline concordance and appropriateness. While this study provides valuable insights, it is limited by the exclusion of certain hospital settings and data constraints. Future research should address these limitations and further investigate the causes of imaging disparities to inform strategies aimed at optimizing patient care.

## Data Availability

Publicly available datasets were analyzed in this study. This data can be found here: https://www.cdc.gov/nchs/nhamcs/documentation/index.html.
