# Supplementary material for: Trends in utilization of imaging among ophthalmic-related emergency department visits in the United States
Source: Front Ophthalmol (Lausanne). 2026 Jan 21;5:1706213. doi: 10.3389/fopht.2025.1706213 (PMC12867896; doi:10.3389/fopht.2025.1706213)
Supplement: Supplementary file 1 [file Table1.docx]

Supplementary Material

# Table S1: National Estimates of Eye ED Visits Involving CT or MRI.

|  | **2016** | **2017** | **2018** | **2019** | **2020** | **2021** | **2022** |
| --- | --- | --- | --- | --- | --- | --- | --- |
| **No. of eye ED visits** | 6,476,047 | 7,372,447 | 5,464,828 | 6,221,806 | 5,539,589 | 4,975,640 | 6,101,618 |
| **No. of eye ED visits involving CT (head)** | 2,445,326 | 2,030,787 | 1,804,115 | 2,110,873 | 2,002,971 | 1,843,877 | 2,412,225 |
| **No. of eye ED visits involving MRI** | 332,588 | 357,870 | 348,723 | 381,106 | 405,230 | 389,095 | 345,153 |
| **No. of eye ED visits involving imaging** | 2,545,867 | 2,126,004 | 1,912,597 | 2,315,981 | 2,170,838 | 1,942,587 | 2,566,826 |
| **Visits involving imaging (%)** | 39.3 | 28.8 | 35.0 | 37.2 | 39.2 | 39.0 | 42.1 |
| **<15** | 2.2 | 6.5 | 4.8 | 4.9 | 2.3 | 3.4 | 2.7 |
| **15≤24** | 7.7 | 4.0 | 9.9 | 6.4 | 10.0 | 3.5 | 6.1 |
| **25≤44** | 20.1 | 25.1 | 21.5 | 26.9 | 18.1 | 18.8 | 15.5 |
| **45≤64** | 35.5 | 38.5 | 27.7 | 30.4 | 31.6 | 37.3 | 28.9 |
| **65≤74** | 16.9 | 10.4 | 16.6 | 17.6 | 13.5 | 16.7 | 18.9 |
| **≥75** | 17.7 | 15.4 | 19.4 | 13.8 | 24.4 | 20.3 | 28.0 |
| **Male** | 41.4 | 37.4 | 40.4 | 36.0 | 40.1 | 42.6 | 39.2 |
| **Female** | 58.6 | 62.6 | 59.6 | 64.0 | 59.9 | 57.4 | 60.8 |
| **White** | 78.3 | 78.7 | 70.1 | 70.6 | 72.6 | 78.8 | 70.9 |
| **Black** | 19.3 | 20.8 | 22.8 | 24.6 | 17.9 | 16.2 | 20.7 |
| **Other** | 2.4 | 0.5 | 7.1 | 4.8 | 9.5 | 5.0 | 8.3 |
| **Northeast** | 13.4 | 12.8 | 15.5 | 15.8 | 17.2 | 19.0 | 16.4 |
| **Midwest** | 30.6 | 28.1 | 18.7 | 19.5 | 22.8 | 18.6 | 18.5 |
| **South** | 33.5 | 43.6 | 38.9 | 42.3 | 35.2 | 40.1 | 45.1 |
| **West** | 22.5 | 15.5 | 26.8 | 22.5 | 24.8 | 22.3 | 19.9 |
| **Private** | 29.4 | 26.9 | 19.9 | 24.1 | 31.0 | 20.1 | 21.6 |
| **Medicare** | 31.4 | 30.5 | 35.8 | 27.5 | 37.4 | 37.5 | 44.8 |
| **Medicaid** | 18.4 | 17.6 | 26.0 | 23.4 | 15.5 | 20.3 | 16.9 |
| **Other** | 20.8 | 25.0 | 18.3 | 25.0 | 16.1 | 22.1 | 16.7 |
| **Weekend** | 76.7 | 79.9 | 70.0 | 76.8 | 76.7 | 79.9 | 71.6 |
| **Weekday** | 23.3 | 20.1 | 30.0 | 23.2 | 23.3 | 20.1 | 28.4 |
| **Admitted** | 84.5 | 79.2 | 84.2 | 87.2 | 76.6 | 80.0 | 85.4 |
| **Not admitted** | 15.5 | 20.8 | 15.8 | 12.8 | 23.4 | 20.0 | 14.6 |
| **Urban** | 84.7 | 84.2 | 91.7 | 85.9 | 91.4 | 92.8 | 87.7 |
| **Rural** | 15.3 | 15.8 | 8.3 | 14.1 | 8.6 | 7.2 | 12.3 |
| **ED Residency** |  |  |  |  |  |  |  |
| **Yes** | 23.8 | 35.9 | 31.8 | 36.2 | 35.5 | 23.1 | 31.5 |
| **No** | 76.2 | 64.1 | 68.2 | 63.8 | 64.5 | 76.9 | 68.5 |
|  |  |  |  |  |  |  |  |

# Table S2: Overall Imaging Use Based on Age.

|  | **2016** | **2017** | **2018** | **2019** | **2020** | **2021** | **2022** |
| --- | --- | --- | --- | --- | --- | --- | --- |
| **No. of eye ED visits involving imaging (<15)** | 54,874 | 137,830 | 92,302 | 114,018 | 50,171 | 66,829 | 68,310 |
| **No. of eye ED visits (<15)** | 663,018 | 739,251 | 668,266 | 701,945 | 465,720 | 491,949 | 516,382 |
| **No. of eye ED visits involving imaging (15-24)** | 196,126 | 84,921 | 189,765 | 147,330 | 216,931 | 67,189 | 155,380 |
| **No. of eye ED visits (15-24)** | 674,791 | 1,028,075 | 724,060 | 644,666 | 774,733 | 432,132 | 615,404 |
| **No. of eye ED visits involving imaging (25-44)** | 512,901 | 534,650 | 412,078 | 623,450 | 393,605 | 364,734 | 397,300 |
| **No. of eye ED visits (25-44)** | 1,972,468 | 2,206,700 | 1,456,975 | 1,681,952 | 1,357,127 | 1,474,928 | 1,579,134 |
| **No. of eye ED visits involving imaging (45-64)** | 903,275 | 819,513 | 530,045 | 704,798 | 685,876 | 724,757 | 741,624 |
| **No. of eye ED visits (45-64)** | 1,865,378 | 2,196,056 | 1,340,318 | 1,882,642 | 1,466,200 | 1,361,341 | 1,583,410 |
| **No. of eye ED visits involving imaging (65-74)** | 429,305 | 221,440 | 316,912 | 407,444 | 293,830 | 324,642 | 484,713 |
| **No. of eye ED visits (65-74)** | 639,268 | 569,927 | 584,665 | 710,409 | 746,668 | 571,079 | 729,838 |
| **No. of eye ED visits involving imaging (≥75)** | 449,386 | 327,650 | 371,494 | 318,941 | 530,423 | 394,436 | 719,498 |
| **No. of eye ED visits (≥75)** | 661,125 | 632,438 | 690,545 | 600,192 | 729,142 | 644,211 | 1,077,450 |

# Table S3: Overall Imaging Use Based on Gender, Race, and Insurance Status.

|  | **2016** | **2017** | **2018** | **2019** | **2020** | **2021** | **2022** |
| --- | --- | --- | --- | --- | --- | --- | --- |
| **No. of eye ED visits involving imaging (male)** | 1,053,315 | 795,713 | 771,819 | 834,655 | 870,404 | 828,353 | 1,006,559 |
| **No. of eye ED visits (male)** | 2,691,958 | 2,850,561 | 2,247,676 | 2,612,099 | 2,201,085 | 2,004,487 | 2,525,608 |
| **No. of eye ED visits involving imaging (female)** | 1,492,552 | 1,330,291 | 1,140,778 | 1,481,326 | 1,300,434 | 1,114,234 | 1,560,267 |
| **No. of eye ED visits (female)** | 3,784,089 | 4,521,886 | 3,217,152 | 3,609,706 | 3,338,504 | 2,971,153 | 3,576,011 |
| **No. of eye ED visits involving imaging (white)** | 1,992,517 | 1,673,605 | 1,341,645 | 1,635,715 | 1,575,572 | 1,531,407 | 1,820,198 |
| **No. of eye ED visits (white)** | 4,859,823 | 5,164,839 | 3,615,488 | 4,259,799 | 3,789,560 | 3,603,338 | 4,292,890 |
| **No. of eye ED visits involving imaging (black)** | 492,283 | 441,921 | 436,019 | 569,884 | 388,621 | 314,097 | 532,515 |
| **No. of eye ED visits (black)** | 1,409,277 | 2,051,455 | 1,578,368 | 1,721,165 | 1,347,380 | 1,164,217 | 1,408,440 |
| **No. of eye ED visits involving imaging (other)** | 61,066 | 10,478 | 134,933 | 110,382 | 206,645 | 97,083 | 214,113 |
| **No. of eye ED visits (other)** | 206,947 | 156,153 | 270,972 | 240,842 | 402,649 | 208,086 | 400,288 |
| **No. of eye ED visits involving imaging (private insurance)** | 749,699 | 571,234 | 381,463 | 558,016 | 673,484 | 390,909 | 553,789 |
| **No. of eye ED visits (private insurance)** | 1,899,441 | 1,851,722 | 1,270,039 | 1,555,146 | 1,334,001 | 1,199,613 | 1,572,859 |
| **No. of eye ED visits involving imaging (Medicare)** | 798,257 | 649,473 | 684,617 | 637,439 | 812,144 | 729,409 | 1,149,876 |
| **No. of eye ED visits (Medicare)** | 1,324,533 | 1,556,272 | 1,303,519 | 1,281,479 | 1,431,125 | 1,213,772 | 1,681,758 |
| **No. of eye ED visits involving imaging (Medicaid)** | 467,406 | 374,583 | 496,925 | 541,702 | 336,082 | 393,519 | 434,949 |
| **No. of eye ED visits (Medicaid)** | 1,845,126 | 2,241,137 | 1,688,484 | 1,757,442 | 1,372,321 | 1,581,982 | 1,685,807 |
| **No. of eye ED visits involving imaging (Other)** | 530,505 | 530,714 | 349,591 | 578,824 | 349,128 | 428,750 | 428,212 |
| **No. of eye ED visits (Other)** | 1,406,947 | 1,723,315 | 1,202,785 | 1,627,738 | 1,402,143 | 980,274 | 1,161,194 |

# Table S4: Overall Imaging Use Based on Hospital Characteristics.

|  | **2016** | **2017** | **2018** | **2019** | **2020** | **2021** | **2022** |
| --- | --- | --- | --- | --- | --- | --- | --- |
| **No. of eye ED visits involving imaging (Urban)** | 2,155,389 | 1,791,110 | 1,753,023 | 1,988,507 | 1,984,225 | 1,802,497 | 2,250,643 |
| **No. of eye ED visits (Urban)** | 5,421,604 | 6,395,652 | 4,924,117 | 5,326,233 | 5,145,716 | 4,523,076 | 5,324,614 |
| **No. of eye ED visits involving imaging (Rural)** | 390,478 | 334,894 | 159,573 | 327,474 | 186,613 | 140,090 | 316,183 |
| **No. of eye ED visits (Rural)** | 1,054,443 | 976,795 | 540,711 | 895,572 | 393,873 | 452,564 | 777,004 |
| **No. of eye ED visits involving imaging (Have EM Residency)** | 549,757 | 684,859 | 587,162 | 788,841 | 744,292 | 424,688 | 791,079 |
| **No. of eye ED visits (Have EM Residency)** | 1,703,357 | 2,224,843 | 2,018,911 | 1,950,579 | 2,186,338 | 1,387,683 | 2,042,459 |
| **No. of eye ED visits involving imaging (No EM Residency)** | 1,762,297 | 1,223,170 | 1,259,997 | 1,388,400 | 1,350,516 | 1,413,109 | 1,717,246 |
| **No. of eye ED visits (No EM Residency)** | 4,312,027 | 4,194,772 | 3,204,737 | 3,935,338 | 3,042,112 | 3,295,877 | 3,901,347 |

# ICD Codes:

A18-, A185, B00-, B005, B02-, B023, B30-, B301, B309, C43-, C431, C44-, C441, C69-, C692, C69-, C696, D18-, D180, D31-, D313, D31-, D316, D32-, D320, D35-, D352, E05-, E050, E10-, E103, E10-, E109, E11-, E113, E119, F08-, F087, F44-, F446, F79-, F79, G20, G20-, G24-, G245, G35, G35-, G43-, G431, G43-, G43B, G45-, G453, G45-, G459, G51-, G510, G51-, G513, G70-, G700, G80-, G809, G90-, G902, G93-, G932, H00-, H000, H00-, H001, H01-, H010, H02-, H020, H02-, H021, H02-, H023, H02-, H024, H02-, H025, H02-, H028, H04-, H040, H04-, H041, H04-, H042, H04-, H045, H05-, H051, H05-, H052, H10-, H100, H10-, H101, H10-, H102, H10-, H103, H10-, H104, H10-, H105, H10-, H108, H11-, H110, H11-, H113, H11-, H115, H11-, H118, H16-, H160, H16-, H161, H16-, H162, H16-, H163, H16-, H164, H17-, H171, H18-, H181, H18-, H182, H18-, H183, H18-, H184, H18-, H185, H18-, H186, H18-, H187, H18-, H188, H20-, H200, H20-, H201, H20-, H202, H21-, H218, H26-, H260, H26-, H261, H26-, H264, H27-, H270, H27-, H271, H30-, H300, H30-, H302, H30-, H309, H31-, H310, H31-, H313, H31-, H314, H33-, H330, H33-, H331, H33-, H332, H33-, H333, H33-, H334, H33-, H338, H34-, H341, H34-, H342, H34-, H348, H35-, H350, H35-, H351, H35-, H352, H35-, H355, H35-, H356, H35-, H357, H35-, H358, H40-, H400, H40-, H401, H40-, H402, H40-, H403, H40-, H404, H40-, H405, H40-, H406, H40-, H408, H42-, H42, H43-, H431, H43-, H432, H43-, H433, H43-, H438, H44-, H440, H44-, H441, H44-, H442, H44-, H445, H46-, H460, H46-, H461, H46-, H463, H46-, H468, H47-, H470, H47-, H471, H47-, H472, H47-, H473, H47-, H474, H47-, H476, H49-, H490, H49-, H491, H49-, H492, H49-, H494, H49-, H498, H49-, H499, H50-, H500, H50-, H501, H50-, H502, H50-, H503, H50-, H504, H50-, H505, H50-, H506, H50-, H508, H50-, H509, H51-, H511, H51-, H512, H51-, H518, H52-, H520, H52-, H521, H52-, H522, H52-, H523, H53-, H530, H53-, H531, H53-, H532, H53-, H534, H53-, H535, H54-, H547, H55-, H550, H55-, H551, H55-, H558, H57-, H570, H57-, H571, H57-, H578, H57-, H579, H59-, H590, H59-, H594, H59-, H598, H81-, H811, H81-, H813, I63-, I639, K52-, K528, L40-, L405, L57-, L570, L82-, L821, L93-, L930, M02-, M023, M05-, M058, M06-, M060, M06-, M068, M06-, M069, M08-, M080, M08-, M082, M31-, M316, M35-, M350, M45-, M459, P04-, P044, P15-, P153, P39-, P391, Q07-, Q078, Q10-, Q100, Q10-, Q103, Q13-, Q131, Q15-, Q150, Q79-, Q796, Q82-, Q825, Q85-, Q850, Q87-, Q874, Q90-, Q90, R42, R42-, R48-, R483, R51-, R510, R51-, R519, R70-, R700, R73-, R730, S00-, S001, S02-, S021, S02-, S023, S02-, S024, S02-, S028, S05-, S050, S05-, S051, S05-, S052, S05-, S053, T15-, T150, T15-, T151, T15-, T158, T26-, T260, T26-, T261, T49-, T495, T50-, T502, T81-, T813, T818, T85-, T853, T86-, T868, Z01-, Z010, Z05-, Z058, Z21-, Z212, Z79-, Z798, Z90-, Z900, Z96-, Z961
